# Safety, pharmacokinetics, and pharmacodynamics of the antisense oligonucleotide RO7239958 in healthy volunteers and adults with chronic hepatitis B infection

**DOI:** 10.1128/aac.00679-25

**Published:** 2025-11-04

**Authors:** Anna Maria Geretti, Alexandre Sostelly, Simon Buatois, Sijie Lu, Annabelle Lemenuel, Gemma Attley, Martin Bopst, Rubén Alvarez-Sánchez, Henrik Mueller, Edward Gane

**Affiliations:** 1 Roche Pharma Research and Early Development, Roche Innovation Centre Basel, Basel, Switzerland; 2 Roche Pharma Research and Early Development, Roche Innovation Center Shanghai 168535 , Shanghai, China; 3 Roche Pharma Research and Early Development, Roche Innovation Center New York 6135 , , Little Falls, New Jersey, USA; 4 Auckland Clinical Studies, Auckland, New Zealand; Providence Portland Medical Center, Portland, Oregon, USA

**Keywords:** hepatitis B virus, cure, PAPD5, PAPD7, TENT4B, TENT4A, locked-nucleic acid, single-stranded oligonucleotide, RO7239958, pharmacokinetics, pharmacodynamics

## Abstract

Poly(A) RNA polymerases D5 and D7 (PAPD5, PAPD7) are host factors that stabilize hepatitis B virus (HBV) RNA transcripts. RO7239958, a liver-targeted locked nucleic acid (LNA) antisense oligonucleotide (ASO), induces intracellular degradation of PAPD5- and PAPD7-encoding messenger RNAs (mRNAs). This first-in-human study evaluated the safety, tolerability, pharmacokinetics (PK), and pharmacodynamics of RO7239958 in healthy volunteers and adults with chronic hepatitis B (CHB) receiving standard-of-care antiviral therapy. Forty healthy volunteers were randomized (4:1) to single ascending subcutaneous doses of RO7239958 (0.1, 0.3, 1.0, or 1.5 mg/kg) or placebo. Based on a population PK model built to predict liver exposure, two parallel CHB cohorts (*n* = 8 and *n* = 7 per cohort) were randomized to two 4-weekly doses of RO7239958 (0.2 or 0.4 mg/kg) or placebo. RO7239958 plasma exposure increased more than dose-proportionally at dose levels of 1.0 and 1.5 mg/kg, suggesting saturation of liver uptake. RO7239958 was generally well tolerated in healthy volunteers; transaminase elevations occurred at the highest doses, including one reversible Grade 3 elevation. The PK model indicated that doses of 0.2 and 0.4 mg/kg would reduce PAPD5 and PAPD7 mRNA by 50% and 62%, respectively, with minimal liver toxicity. Consistent with predictions, RO7239958 was well-tolerated and safe in patients. At 0.4 mg/kg, the maximum reduction from baseline in serum hepatitis B surface antigen was observed on Day 43 (mean decline 0.2 log_10_ IU/mL; range 0.0–0.5 log_10_ IU/mL). While RO7239958 showed a potentially narrow therapeutic window, PAPD5 and PAPD7 remain potential therapeutic targets in HBV curative strategies.

**CLINICAL TRIALS:**

This study was registered at NCT03762681.

## INTRODUCTION

Hepatitis B virus (HBV) establishes persistent infection as a covalently closed circular DNA (cccDNA) in the nucleus of infected hepatocytes, using host transcription factors to support the expression of viral proteins (1–3). Major RNA transcripts produced from cccDNA include pregenomic RNA (pgRNA), which is the template for reverse transcription that synthesizes genomic HBV DNA and also codes for core and polymerase proteins; precore messenger RNA (mRNA), which expresses the precore protein that becomes hepatitis B e antigen (HBeAg); and the subgenomic mRNAs, which code for hepatitis B surface antigen (HBsAg) (4, 5). HBV DNA fragments that do not encode the complete viral genome commonly integrate into the host genome and serve as a source of HBsAg production (6–10). High levels of HBsAg expression are thought to play a role in facilitating HBV persistence by suppressing antiviral immune responses (11–15). Seroclearance of HBsAg is a key endpoint in assessing functional cure of the infection and is associated with improved clinical outcomes (16). However, HBsAg loss is rarely attained with standard-of-care nucleos(t)ide analogs (NAs), which block HBV DNA synthesis from pgRNA by acting on the viral polymerase enzyme (2, 16). New treatments are being researched to achieve a functional cure of chronic HBV infection (2, 17).

The non-canonical poly(A) RNA polymerase-associated domain-containing proteins 5 and 7 (PAPD5 and PAPD7, also called TENT4B and TENT4A, respectively) are host factors used by HBV to stabilize mRNA transcripts and maintain HBV gene expression (3, 18–21). PAPD5 and PAPD7 are involved in the generation of long, guanylated poly(A) mRNA tails that protect mRNA from rapid deadenylation and decay (3, 19). *In vitro* studies have shown that transcripts encoding secreted proteins are relatively frequently guanylated and sensitive to PAPD5 and PAPD7 depletion (19). Consistent with these observations, we previously showed that the suppression of PAPD5 and PAPD7 using liver-targeted LNA SSOs or via treatment with the small-molecule inhibitor RG7834 reduced HBsAg levels in HBV-infected primary hepatocytes and liver cell lines in a dose-dependent fashion, without affecting cell viability (highest tested concentration 20 µM and 1 µM, respectively) (3). Dose-dependent reductions in intracellular and serum HBsAg levels were also demonstrated in HBV-infected human liver chimeric urokinase-type plasminogen activator/severe combined immunodeficiency mice (3, 18, 22). In this mouse model, inhibition of PAPD5 and PAPD7 also led to reductions in serum HBV DNA, HBeAg, and intracellular core protein albeit to a lesser extent than observed for HBsAg (22). Preferential guanylation may, therefore, render subgenomic HBsAg-encoding mRNA especially sensitive to PAPD5- and PAPD7-mediated regulation (3, 19, 22).

Previous preclinical studies with RG7834 revealed a risk of neurotoxicity (polyneuropathy) with chronic dosing (23). RO7239958 is a novel liver-targeted HBV gene expression inhibitor that uses a locked nucleic acid (LNA)-containing single-stranded antisense oligonucleotide (ASO) to induce intracellular degradation of mRNAs encoding PAPD5 and PAPD7. RO7239958 targets a conserved sequence shared by PAPD5 and PADP7 mRNAs and inhibits both transcripts equally. To achieve hepatocyte-specific targeting, RO7239958 is conjugated to N-acetylgalactosamine (GalNAc), a high-affinity ligand of the asialoglycoprotein receptor (ASGPR), which is predominantly expressed on the hepatocyte cell surface (24, 25). After ASGPR-mediated endocytosis, the GalNAc-conjugated LNA-ASO is released; degradation of GalNAc occurs without affecting the LNA-ASO sequence (25, 26). After intracellular release, the LNA-ASO hybridizes with PAPD5 and PAPD7 mRNAs, triggering RNAseH-mediated degradation. ASGPR-mediated uptake processes are saturable, and levels of RO7239958 above which ASGPR saturation occurs are expected to result in proportionally lower liver uptake and increased plasma and kidney drug concentrations (27, 28). Preclinical data in rodents and cynomolgus macaques indicate that plasma and urine levels of GalNAc-conjugated LNA-ASOs are indicators of ASGPR saturation, with a shift to supra-dose proportional plasma kinetics and appearance of the drug in the urine as ASGPR liver uptake begins to saturate with increasing dose (29). Dosing of GalNAc-conjugated LNA-ASOs like RO7239958 requires, therefore, careful modulation based on plasma and urine pharmacokinetics (PK) to optimize hepatocyte targeting while minimizing unnecessary extra-hepatic exposure (27, 29–31). For RO7239958, preclinical PK and toxicity studies in rodents and macaques confirmed predominant hepatic and renal distribution, negligible uptake, and no target knockdown in peripheral nerve tissue and no relevant treatment-related findings outside the liver and kidney (rodent only).

Here, we report the results of the first-in-human study designed to evaluate the safety, tolerability, and PK of a range of RO7239958 doses in healthy volunteers, and the safety, tolerability, PK, and pharmacodynamics (PD) of RO7239958 in patients with chronic hepatitis B infection (CHB) who were NA-treated and virologically suppressed.

## RESULTS

### Study design

**Fig 1:**
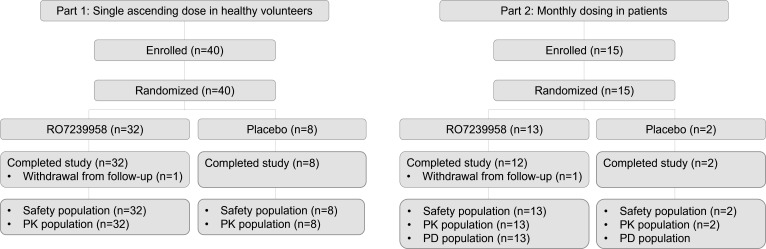
Study design and flow.

This randomized, observer-blind, placebo-controlled, adaptive phase 1 study was conducted in healthy volunteers (Part 1) and adults with CHB who were receiving NA therapy stably and had no significant liver fibrosis (Part 2) (Fig. 1).

### Baseline characteristics

#### Part 1. Healthy volunteers

In Part 1, four cohorts of healthy volunteers (*n* = 10 per cohort) received a single subcutaneous dose of RO7239958 (0.1, 0.3, 1.0, or 1.5 mg/kg) or placebo at a 4:1 ratio. The four dose groups were well-matched for demographic and baseline characteristics (Table 1). Most participants were male (38/40) and White (26/40).

**TABLE 1:** Demographic and baseline characteristics of healthy volunteers (Part 1 of the study)

Characteristics		Placebo (*n* = 8)	Cohort 1 0.1 mg/kg (*n* = 8)	Cohort 2 0.3 mg/kg (*n* = 8)	Cohort 3 1.0 mg/kg (*n* = 8)	Cohort 4 1.5 mg/kg (*n* = 8)	All cohorts (*n* = 40)
Age, mean (SD) years		37 (13)	37 (15)	29 (8)	32 (14)	29 (7)	33 (11)
Male, *n* (%)		7 (87.5)	8 (100)	7 (87.5)	8 (100)	8 (100)	38 (95.0)
Ethnicity, *n* (%)	White	5 (62.5)	7 (87.5)	3 (37.5)	5 (62.5)	6 (75.0)	26 (65.0)
	Asian	2 (25.0)	1 (12.5)	2 (25.0)	1 (12.5)	0	6 (15.0)
	Other^*a*^	1 (12.5)	0	3 (37.5)	2 (25.0)	2 (25)	8 (20.0)
Weight, mean (SD) kg		82.6 (15.7)	84.2 (6.9)	75.7 (9.4)	83.0 (8.4)	74.0 (9.9)	79.9 (15.3)
BMI, mean (SD) kg/m^2^		25.9 (4.4)	26.1 (1.6)	24.6 (3.6)	25.6 (2.6)	23.2 (3.2)	25.1 (3.3)

^*a*^ Black or African American, Native Hawaiian or other Pacific Islander, or multiple ethnicities. BMI, body mass index; SD, standard deviation.

#### Part 2. Participants with CHB

In Part 2, 15 patients were randomized to two parallel cohorts, comprising eight and seven participants, respectively. In the first cohort, all participants received two subcutaneous doses of RO7239958 (0.2 mg/kg) at an interval of 4 weeks between doses. In the second cohort, five participants received two subcutaneous doses of RO7239958 (0.4 mg/kg) at an interval of four weeks between doses, whereas two received placebo. All participants were male, and most were White (7/15) or Asian (5/15). All were on stable NA therapy, usually with tenofovir disoproxil fumarate (10/15), and all had suppressed HBV DNA levels as per the eligibility criteria. The two parallel arms were well balanced for baseline HBsAg levels (mean 3.5 log_10_ IU/mL [range 2.8–4.2]). A total of 3/15 participants had a positive HBeAg test (Table 2).

**TABLE 2:** Demographic and baseline characteristics of adults with chronic hepatitis B (Part 2 of the study)

Characteristics		RO7239958 dose level		Placebo (*n* = 2)
		0.2 mg/kg (*n* = 8)	0.4 mg/kg (*n* = 5)	
Age, mean (SD) years		48 (7)	46 (7)	46 (18)
Male, *n* (%)		8 (100)	5 (100)	2 (100)
Ethnicity, *n* (%)	White	3 (37.5)	4 (80.0)	0
	Asian	4 (50.0)	1 (20.0)	0
	Other^*a*^	1 (12.5)	0	2 (100.0)
Weight, mean (SD) kg		80.9 (10.9)	77.4 (6.6)	82.2 (5.0)
BMI, mean (SD) kg/m^2^		26.1 (2.3)	26.1 (1.8)	25.8 (2.6)
HBeAg status, *n* (%)	Negative	5 (62.5)	5 (100.0)	2 (100.0)
	Positive	3 (37.5)	0	0
HBsAg, mean (SD) log_10_ IU/mL		3.6 (0.5)	3.3 (0.5)	3.8 (0.5)
ALT, mean (SD) U/L		25.1 (12.3)	24.1 (9.5)	22.7 (1.4)
AST, mean (SD) U/L		24.6 (4.0)	22.5 (2.9)	24.5 (0.7)
NUC therapy, *n* (%)	Tenofovir	5 (62.5)	4 (80.0)	1 (50.0)
	Entecavir	3 (37.5)	1 (20.0)	1 (50.0)

^*a*^ Black or African American, Native Hawaiian or other Pacific Islander, or multiple ethnicities. ALT, alanine transaminase; AST, aspartate aminotransaminase; BMI, body mass index; CHB, chronic hepatitis B; HBeAg, hepatitis B e-antigen; HBsAg, hepatitis B surface antigen; HBV, hepatitis B virus; NUC, nucleos(t)ide analog; SD, standard deviation.

### Safety and tolerability in healthy volunteers

All volunteers received one dose of study medication. During follow-up, one volunteer withdrew consent. There were no serious adverse events (SAEs) and no AEs that led to withdrawal, modification, or interruption of study treatment. AEs were reported in 19/32 volunteers who received RO7239958 and 7/8 of the placebo participants. With the exception of three volunteers who had grade 3 AEs of elevated transaminases, all AEs reported were grade 1. One of the grade 3 AEs occurred in the placebo group and was characterized by elevated AST levels exceeding ALT levels (ALT > AST), along with elevated creatinine kinase (CK); this was judged to be related to strenuous exercise. The other two events occurred in the cohort receiving RO7239958 at the highest dose level (1.5 mg/kg); one presented with a similar pattern to the event observed in the placebo participant and was similarly related to strenuous exercise. The second event was considered related to the study treatment. It occurred in a 24-year-old White male with a BMI of 23.3 kg/m^2^. The results of investigations performed on this participant are summarized in Table S1. The ALT was mildly elevated pre-dosing (64 U/L), increased gradually post-dosing, reaching a peak of 355 U/L (7.9× ULN) on study Day 31, and subsequently declined. The AST and ALP showed more minor parallel elevations, peaking at 80 U/L (1.8× ULN) and 168 U/L (1.5× ULN), respectively. Bilirubin and GGT levels remained within normal limits. Serum copper was 8 µmol/L on Day 50 (reference range 11.8–22.8 µmol/L), and this finding, alongside a history of childhood seizures, prompted a liver biopsy on Day 65. Findings of the biopsy excluded Wilson’s disease and diagnosed a mild steatohepatitis.

Smaller transient grade 1 elevations of ALT in participants receiving RO7239958 with ALT > AST occurred in 4/8 participants in cohort 3 (1.0 mg/kg) and 2/8 participants in cohort 4 (1.5 mg/kg). With the exception of the mild increase in ALP noted above, no changes in liver function were observed in any of the participants. No hematological, renal function, or urinary abnormalities were observed in the four healthy volunteer cohorts, and RO7239958 had no effects on ECG or vital signs.

### Pharmacokinetics in healthy volunteers

**Fig 2:**
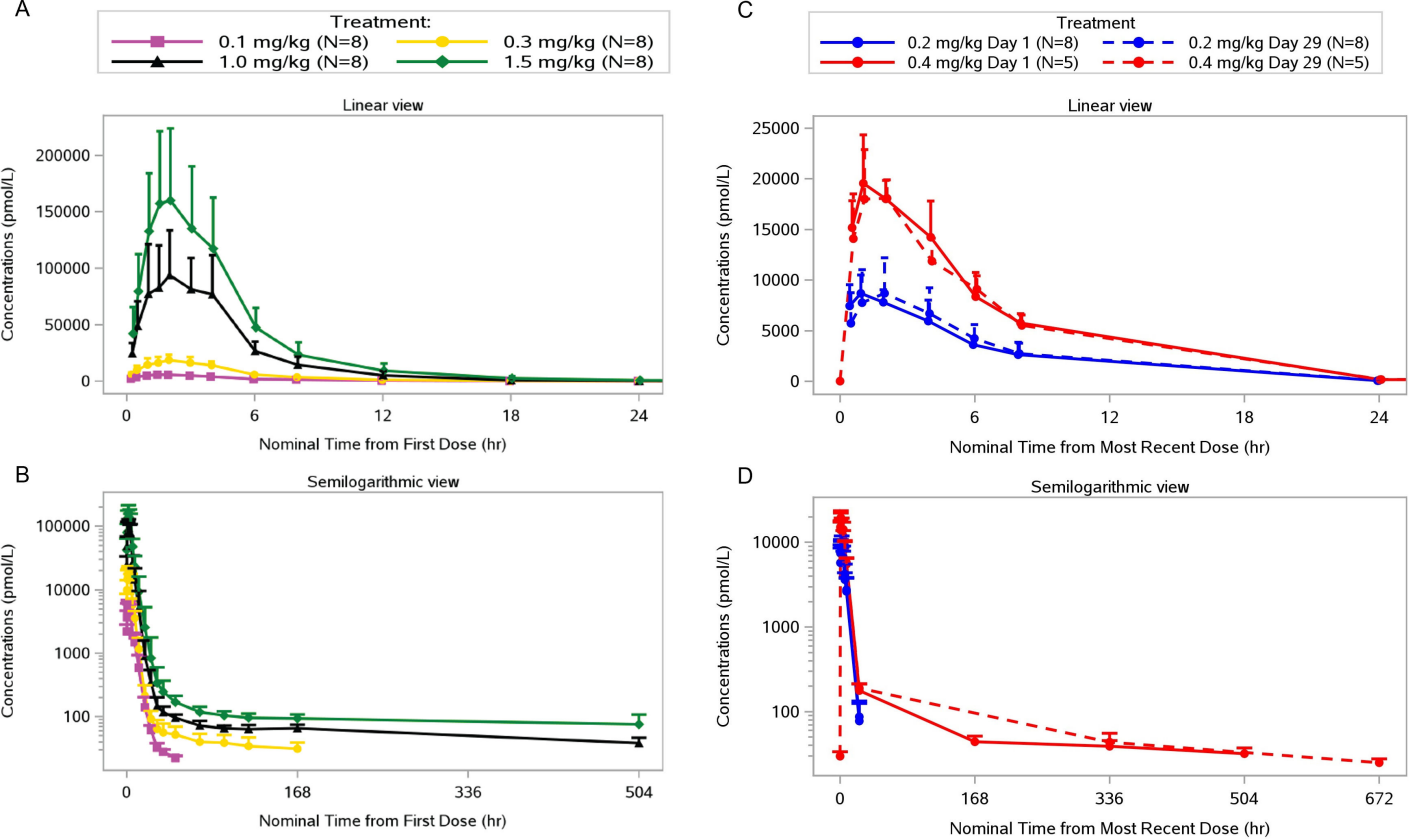
Mean plasma concentration-time profile of RO7239958 in healthy volunteers (**A, B**) and adults with chronic hepatitis B (**C, D**) over 24 h (**A, C**), 504 h (**B**), and 672 h (**D**) after dosing.

**Fig 3:**
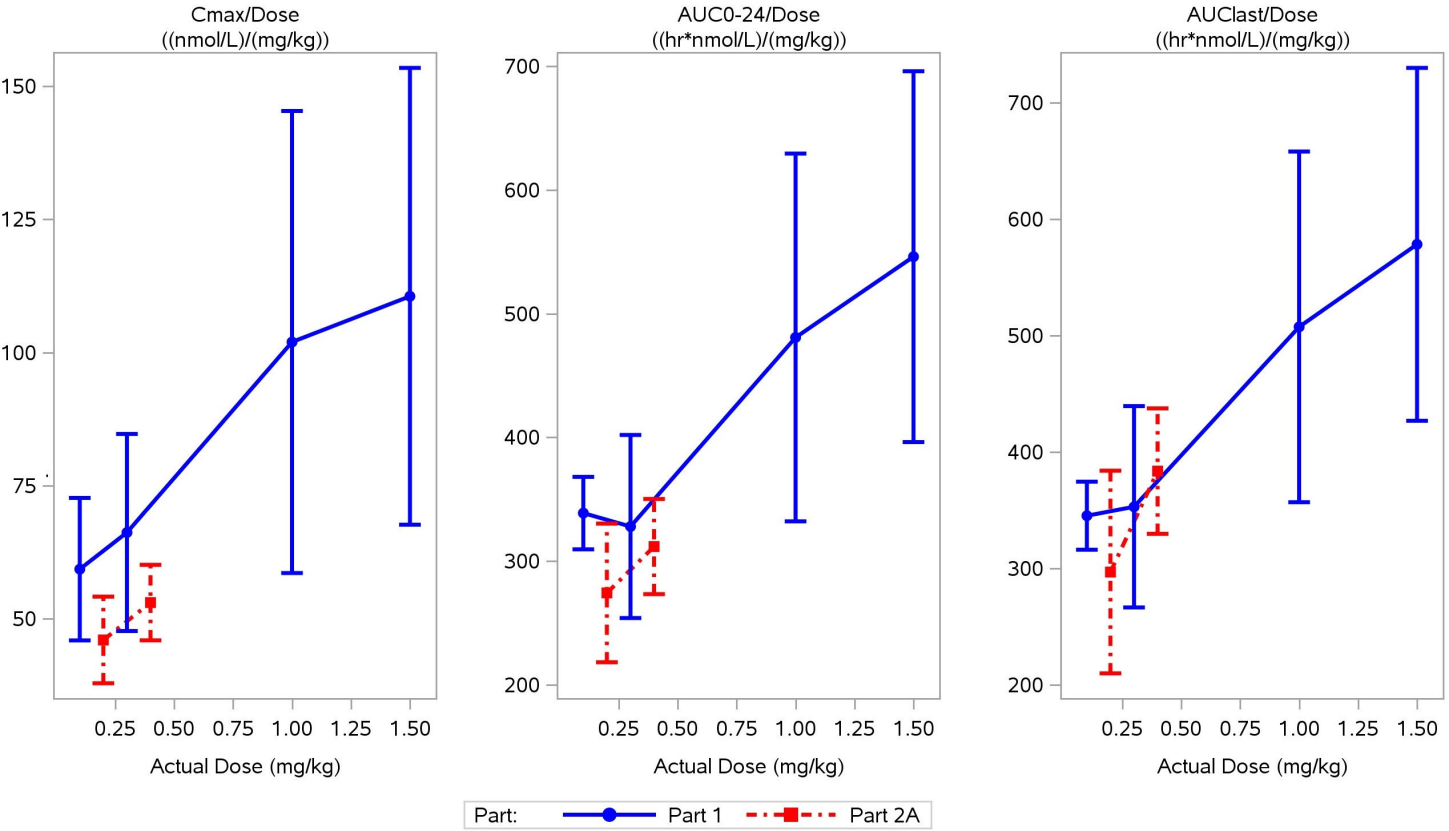
Mean exposure of RO7239958 vs dose in healthy volunteers (Part 1) and adults with chronic hepatitis B (Part 2). Error bars indicate standard deviation. Abbreviations: AUC_0–24_, area under concentration-time curve to 24 h post-dose; AUC_last_, area under concentration-time curve to the last measurable point; and *C*_max_, maximum plasma concentration.

In healthy volunteers, RO7239958 appeared rapidly in plasma (median *t*_max_ 1–2 h, range 1.5–4 h) and a biphasic elimination time course was observed with most of the drug cleared from plasma during a distribution phase of up to 48 h, followed by a terminal elimination phase with a longer *t*_1/2_ (Fig. 2A and B). The latter may represent drug redistributed from tissue. In healthy volunteers, the plasma exposure of RO7239958 increased in a slightly more than dose-proportional manner at dose levels of 1 and 1.5 mg/kg (Fig. 3). Across the four volunteer cohorts, the mean fraction of RO7239958 dose recovered in urine over 24 h was approximately 0.2% to 0.4%, indicating that saturation of liver uptake was not reflected in urine. Most urinary excretion of RO7239958 occurred within 12 h of dosing.

### Modeling, simulation, and dose selection for use in participants with CHB

At the time of selecting the dose for Part 2, the PK data set comprised data from 32 healthy volunteers after single doses of RO7239958 ranging from 0.1 to 1.5 mg/kg. Evaluation of plasma kinetics indicated that the shift to supra-dose proportionality would occur within the 0.4 to 1 mg/kg range. According to preclinical data and literature data on other GalNAc-conjugated molecules, this shift was assumed to be associated with a saturation of liver uptake process via the ASGPR transporter. For this reason, transport from the plasma to the liver was modeled with Michaelis-Menten kinetics where *V*m/*F* was estimated at 21.9 mg/h and *K*m was estimated at 0.622 µg/mL. The quality of the goodness-of-fit plots and the adequate precision of the parameter estimates indicated that the model effectively described the PK profiles for RO7239958. Based on the occurrence of transaminase elevations at dose levels of 1.0 and 1.5 mg/kg in healthy volunteers, the corresponding predicted median liver exposure at *C*_max_ after a single dose of 1.0 mg/kg was considered the threshold below which exposure could be targeted. Following two doses of RO7239958 at 0.2 or 0.4 mg/kg at an interval of 4 weeks, RO7239958 liver concentrations were predicted to remain below this threshold in more than 95% of patients throughout the dosing interval (Fig. S1). The choice of these two doses was, therefore, supported by the simulations.

### Pharmacokinetics in participants with CHB

The PK profile and parameters in participants with CHB were comparable to those seen in healthy participants. In patients, RO7239958 exposure did not deviate from proportionality up to 0.4 mg/kg (Fig. 3). No evidence of plasma RO7239958 accumulation was observed after two doses of RO7239958 at either 0.2 mg/kg or 0.4 mg/kg (Fig. 2C and D). Mean exposure (*C*_max_ and AUC) and the percentage of RO7239958 recovered in urine were similar on the two dosing days (Days 1 and 29).

### Safety and tolerability in participants with CHB

All participants received the two planned doses of study medication. There were no SAEs and no AEs that led to withdrawal, modification, or interruption of study treatment in participants with CHB. AEs were reported in one of two placebo participants and 5 of 13 participants receiving RO7239958, comprising 4/8 and 1/5 in the 0.2 mg/kg and 0.4 mg/kg cohorts, respectively. Three treatment-related AEs were reported in two patients receiving RO7239958: one grade 1 injection site reaction (0.2 mg/kg cohort) and one grade 1 fatigue and dizziness (0.4 mg/kg cohort); all resolved without treatment. In total, four patients had transient grade 1 increases in AST (one on placebo and two receiving RO7239958 0.2 mg/kg) and/or ALT (one receiving RO7239958 0.2 mg/kg and one receiving RO7239958 0.4 mg/kg). None were considered related to treatment. All other parameters of liver function were within normal range for all patients. No hematological, renal function, or urinary abnormalities were observed, and RO7239958 had no effects on ECG and vital signs.

### Pharmacodynamics

**Fig 4:**
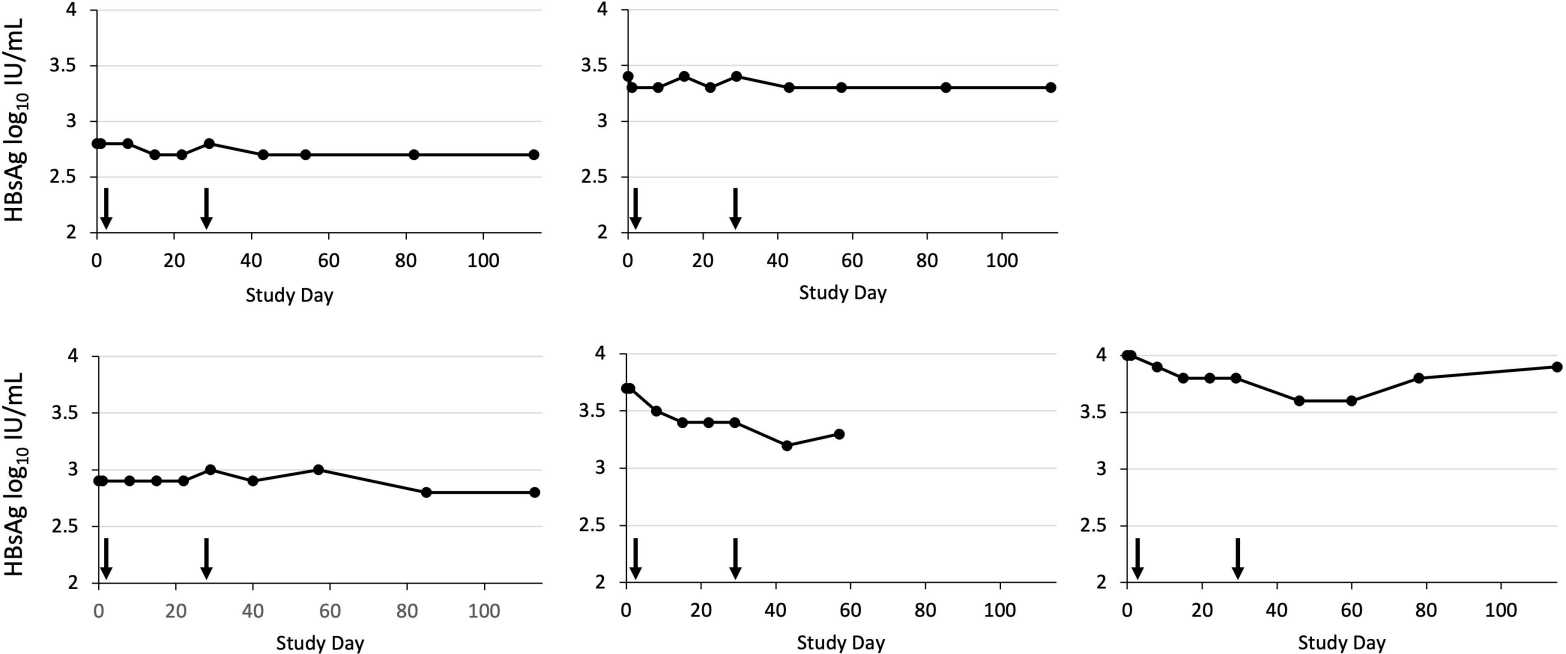
Serum HBsAg levels in five participants with chronic hepatitis B following administration of two 4-weekly doses of RO7235598 0.4 mg/kg (indicated by the arrows).

HBsAg levels showed limited changes among all participants (Table 3). The mean maximum HBsAg change was +0.01 [± 0.1] log_10_ IU/mL in the two placebo participants and −0.01 [± 0.06] log_10_ IU/mL in the eight patients who received RO7239958 0.2 mg/kg. The five patients who received RO7239958 0.4 mg/kg had a mean maximum change of −0.20 (± 0.22 log_10_) IU/mL, which occurred around Day 43 (Fig. 4). Among the five participants in this cohort, changes in HBsAg levels (in log_10_ IU/mL) between Day 1 (pre-dose) and Day 43 (2 weeks post last dose) were as follows: (i) 2.8 to 2.7 (−0.1); (ii) 3.4 to 3.3 (−0.1); (iii) 4.0 to 3.6 (−0.4); (iv) 2.9 to 2.9 (−0.0); (v) 3.7 to 3.2 (−0.5) (Table 3). HBV DNA suppression was maintained throughout the study with no instances of confirmed virological breakthrough. Two of the three patients with HBeAg showed a modest decrease in HBeAg levels (from 1.1 to 0.8 IU/mL and from 0.9 to 0.8 IU/mL, respectively), with no HBeAg seroconversions (not shown).

**TABLE 3:** HBsAg levels (in log_10_ IU/mL) in participants with chronic hepatitis B who received two doses of RO7239958 4 weeks apart

Participant^*a*^	Placebo		0.2 mg/kg arm								0.4 mg/kg arm				
	33/M/B	59/M/O	49/M/A*	43/M/O*	45/M/A*	34/M/W	57/M/W	56/M/A	50/M/W	46/M/A	57/M/W	45/M/W	43/M/W	37/M/W	46/M/A
Study day															
Baseline	4.2	3.4	3.6	3.2	3.1	4.2	4.3	2.8	3.9	3.9	2.8	3.3	4.0	2.9	3.7
Day 1	4.2	3.5	3.6	3.2	3.1	4.2	4.2	2.8	3.9	3.8	2.8	3.4	4.0	2.9	3.7
Day 8	4.2	3.4	3.6	3.2	3.1	4.2	4.2	2.9	4.0	3.9	2.7	3.3	3.9	2.9	3.5
Day 15	4.2	3.6	3.5	3.2	3.1	4.2	4.2	2.8	3.9	3.8	2.7	3.4	3.8	2.9	3.4
Day 22	4.2	3.4	3.6	3.2	3.1	4.2	4.2	2.8	3.9	3.9	2.7	3.3	3.8	2.9	3.4
Day 29	4.2	3.4	3.6	3.3	3.1	4.2	4.2	2.8	3.9	3.9	2.8	3.4	3.8	3.0	3.4
Day 43	4.2	3.4	3.5	3.2	3.2	4.2	4.2	2.7	3.9	4.0	2.7	3.3	3.6	2.9	3.2
Day 57	4.2	3.4	3.5	3.2	3.0	4.1	4.2	2.8	3.9	3.9	2.7	3.3	3.6	3.0	3.3
Day 85	4.2	3.4	3.5	3.1	3.0	4.2	4.2	2.8	3.9	3.8	2.7	3.3	3.8	2.8	NA
Day 113	NA	NA	3.5	3.1	3.0	4.1	4.1	2.7	NA	NA	2.7	3.3	3.9	2.8	NA

^*a*^ Shown by age, sex (M, male) and ethnicity (B, Black; O, Other; A, Asian; W, White); (*) indicates those who tested positive for HBeAg. NA, not available.

### Immunogenicity

In Part 1, all healthy volunteers were negative for antidrug antibodies (ADAs) at baseline. The post-baseline incidence of positive ADAs among RO7239958 participants was 5/40 (12.5%), comprising two individuals in the 1.0 mg/kg cohort and three in the 1.5 mg/kg cohort, respectively, on Day 22. In Part 2, all participants with CHB were negative for ADAs at baseline. The post-baseline incidence of ADAs was 2/15 (13.3%), comprising two individuals in the 0.4 mg/kg cohort at both Day 29 (post last dose) and Day 57 (4 weeks post last dose). ADA titers were all <10 ng/mL, with most in the 1 to 3 ng/mL range and, therefore, considered weakly positive. ADA detection did not correlate with safety events or abnormal PK findings.

## DISCUSSION

In this two-part study, we investigated single doses of RO7239958 between 0.1 and 1.5 mg/kg in healthy volunteers and two doses of RO7239958 of 0.2 or 0.4 mg/kg given at a 4 week interval in adults with CHB who showed virological suppression on NA therapy. Whereas treatment was overall well tolerated with no serious AEs, transaminase elevations with ALT > AST were observed in healthy volunteers who received the highest single doses of RO7239958 (1.0 mg/kg and 1.5 mg/kg). No such trends were observed with the lower dose levels (0.2 and 0.4 mg/kg) administered to patients with CHB in Part 2 of the study. While safe and well tolerated in this group, two doses of RO7239958 at 0.2 mg/kg or 0.4 mg/kg administered four weeks apart had a negligible to modest HBsAg-lowering effect.

Preclinically, a comprehensive non-clinical safety evaluation in rats and macaques, along with plasma and tissue distribution analyses, confirmed the predominant hepatic uptake of the GalNac-conjugated LNA-ASO used in this study, with minimal exposure in non-hepatic organs (except the kidney), and in particular with negligible peripheral nerve exposure and no target knockdown supporting a low risk of on- or off-target toxicity beyond the liver. Peripheral neurotoxicity has been reported in animal studies using oral small-molecule inhibitors of PAPD5 and PAPD7, raising concerns about the safety of this target class (23, 32). We did not observe peripheral neurotoxicity in shorter term and chronic toxicology studies in rodents or non-human primates and the short-term human exposures reported here. Several factors may account for this difference. The PK/PD profiles of ASOs differ from those of small molecules, achieving targeted intracellular engagement and lower peak systemic concentrations. Notably, RO7239958 exhibits preferential hepatic uptake, which limits systemic and thus peripheral nerve exposure and reduces the likelihood of on- or off-target effects in neuronal tissues.

At the highest dose level of RO7239958 (1.5 mg/kg), one healthy volunteer had a treatment-related grade 3 ALT elevation (up to 7.9 × ULN) that began on Day 22 after dosing and was gradually improving but had not yet fully resolved at study completion (Day 85). The ALT increase was accompanied by a transient increase in ALP, whereas bilirubin levels remained within normal limits. Detailed investigations excluded other incident etiologies, indicating that the ALT flare should be considered indicative of potential drug-induced liver toxicity (33, 34). The presence of a mildly raised ALT at study entry and findings from a liver biopsy performed on Day 65 suggested a possible contributing role of potentially pre-existing liver steatosis. However, more minor transient ALT elevations were also seen in 4/8 healthy volunteers in cohort 3 (1.0 mg/kg) and 2/8 healthy volunteers in cohort 4 (1.5 mg/kg). Liver findings with oligonucleotides (single-stranded ASOs as well as siRNAs) are well known from animal studies albeit with limited translation to humans (35–37). Typical findings consist of basophilic granules in the cytoplasm of hepatic Kupffer cells and hepatocytes, hepatocellular vacuolation, single cell necrosis, and inflammatory infiltrates, mainly observed in rodents (35–37). Our observations confirm that the potential hepatotoxicity of LNA-ASOs requires careful evaluation.

The PK of RO7239958 was consistent with predictions from preclinical models and similar to that of other GalNAc-conjugated LNA-ASOs (29). As the dose increases, plasma clearance of LNA-ASOs can decrease due to saturation of liver uptake via ASGPR (29, 38). In healthy volunteers, RO7239958 AUC and *C*_max_ increased in a supra-dose proportional manner at dose levels of 1.0 and 1.5 mg/kg, suggesting that ASGPR saturation had begun to occur. However, <1% of the RO7239958 1.5 mg/kg dose was excreted in the urine of volunteers over the 24 h collection period, and there was no evidence of an adverse effect of RO7239958 on renal function, indicating a minimal level of ASGPR saturation and extra-hepatic drug exposure at this dose level. Plasma and urine PK in participants with CHB were similar to those measured in healthy volunteers, with no evidence of plasma accumulation after two 4-weekly doses of 0.2 or 0.4 mg/kg.

Based on primate modeling data, a RO7239958 liver concentration of 500 pmol/g was expected to provide around 50% of maximal PAPD5 and PAPD7 mRNA knockdown (IC_50_). Furthermore, liver concentrations between 6- and 20-fold above this IC_50_ value were expected to be required for producing a sufficient mRNA knockdown to induce a robust effect on HBsAg expression. To achieve this target hepatic concentration range, doses between 0.15 and 0.5 mg/kg/week at steady state were predicted to be required (Data on file). A population PK model was built using data from healthy volunteers to predict liver exposure in patients and to guide the selection of a dose that would reduce the risk of liver toxicity while preserving efficacy. The safety of the two doses selected was confirmed, with no evidence of toxicity after two 4-weekly doses.

Preclinical studies indicated that the mRNA-destabilizing effect of PAPD5 and PAPD7 depletion or inhibition is HBV-selective, with the expression of host mRNAs only moderately affected (19, 20, 22). Subgenomic mRNA transcripts encoding HBsAg appear to be more susceptible to PAPD5 and PAPD7 depletion than pregenomic and precore RNA, from which other viral antigens are derived (3, 22). RO7239958-induced PAPD5 and PAPD7 knockdown would, therefore, be expected to have a preferential HBsAg-lowering effect. In participants with CHB, reductions in HBsAg were overall modest. After two doses of 0.4 mg/kg at an interval of 4 weeks, two of five participants showed an HBsAg decline of 0.5 and 0.4 log_10_ IU/mL at Day 43. In at least one participant, this was followed by a rebound, suggesting a possible, transient therapeutic effect. These observations were in contrast with the stability of HBsAg levels in placebo participants and participants in the 0.2 mg/kg cohort. Interpretation of the findings requires caution due to the small sample size. Nonetheless, the magnitude of the effect does not compare favorably with results obtained in clinical trials of RNA therapeutics directly targeting HBV RNA transcripts (29, 39–43). Compared with the mean nadir decline of 0.2 log_10_ IU/mL on Day 43 after two 4-weekly doses of RO7239958 0.4 mg/kg, compounds directly targeting HBV RNA transcripts can induce reductions in HBsAg ≥1 log_10_ IU/mL (39, 44) and achieve HBsAg loss in a subset of participants (43, 45). Interestingly, recent studies have shown greater HBsAg responses among participants with baseline HBsAg levels < 3.5 log_10_ IU/mL; however, we were unable to assess this effect in our study because only patients with baseline HBsAg levels ≥ 2.4 log_10_ IU/mL were enrolled (43). The largest HBsAg reductions with RO7239958 0.4 mg/kg (0.4 and 0.5 log_10_ IU/mL) were measured in two participants with baseline HBsAg levels of 4.0 and 3.7 log_10_ IU/mL, respectively. Doses of RO7239958 above 0.4 mg/kg may have achieved greater reductions in HBsAg; however, the ability to dose-escalate was limited by ASGPR saturation and transaminase elevations observed in healthy volunteers at the 1.0 and 1.5 mg/kg doses. These observations point to a narrow therapeutic window for RO7239958.

A more extended treatment duration and/or more frequent dosing may have resulted in greater PD responses. However, a decision was made to terminate the study based on the risk-benefit evaluation, whereby increasing the efficacy might have come at the cost of a risk of hepatotoxicity. Although RO7239958 did not produce the desired HBsAg-lowering effect in our study, PAPD5 and PAPD7 remain potential therapeutic targets of interest in the search for an HBV cure.

### Conclusions

Two 4-weekly doses of RO7239958 at either 0.2 mg/kg or 0.4 mg/kg in participants with CHB who were receiving NA therapy were well tolerated and safe. Modest reductions in HBsAg of up to 0.5 log_10_ IU/mL were observed after two 4-weekly doses of 0.4 mg/kg. In light of indications of ASGPR saturation and potential liver toxicity with higher doses of RO7239958 in healthy volunteers, there was limited scope for dose escalation in patients. Although the clinical development of RO7239958 has been discontinued, PAPD5 and PAPD7 remain potential therapeutic targets for inhibiting HBV expression.

## MATERIALS AND METHODS

### Study design

The primary objective of the study was to evaluate the safety and tolerability of RO7239958 after single subcutaneous ascending doses in healthy volunteers and after two subcutaneous doses at two different dose levels in patients. Plasma and urine PK were assessed as secondary objectives. Part 2 of the study included evaluating the PD effects of RO7239958 as an additional secondary objective. An exploratory objective of the study was to evaluate the relationship between RO7239958 dose and plasma exposure.

Part 1 was conducted at one center in New Zealand. Four cohorts of healthy volunteers (*n* = 10 per cohort) received a single dose of RO7239958 or placebo at a ratio of 4:1. Four ascending dose levels of RO723995 were evaluated: 0.1, 0.3, 1.0, or 1.5 mg/kg. Sentinel dosing was performed, whereby two volunteers from each cohort (including ≥1 who received RO7239958) were followed for 24 h after dosing to monitor for acute reactions, prior to dosing the remaining volunteers. Safety was monitored for at least 28 days after dosing in each cohort, and all available safety and PK data were reviewed prior to each dose escalation. Part 2 was conducted at seven centers across Bulgaria, Hong Kong, Korea, New Zealand, and the United Kingdom. Fifteen patients were randomized to two parallel cohorts that received two doses of RO7239958 at an interval between doses of 4 weeks and at a dose level of 0.2 mg/kg (cohort 1) or 0.4 mg/kg (cohort 2). According to the study protocol, patients in each arm were to be randomized 6:1 to RO7239958 or placebo, but a randomization error resulted in allocation of the two placebo assignments to cohort 2; thus, all eight patients in cohort 1 received RO7239958 0.2 mg/kg and five patients in cohort 2 received RO7239958 0.4 mg/kg. Study drugs were administered by investigators or nurses at the study clinic in the fasted state either 2 h before or 2 h after a meal. Volunteers and patients were followed up until 12 weeks (84 days) after the last dose of study drug.

The study was performed in accordance with the International Conference on Harmonization E6 Good Clinical Practice guidelines and the principles of the Declaration of Helsinki, or the laws and regulations of the country in which the research was conducted. The study was approved by the institutional review board/ethics committee of the participating sites. Written informed consent was obtained from all participants.

### Participants

Male and female healthy volunteers and adults with CHB aged 18–65 years and weighing 45–150 kg (body mass index [BMI] 18–32 kg/m^2^) were eligible to participate in the study. Females were required to be of non-childbearing potential. In Part 1, eligible healthy volunteers were defined as non-smokers with no evidence of clinically relevant past medical history, no active or chronic disease (as judged by the Investigator) and not likely to need concomitant medication during the study period. In Part 2, eligible participants with CHB had a detectable serum HBsAg for >6 months before screening, were receiving entecavir or tenofovir treatment for >3 months prior to randomization, were expected to remain on treatment throughout the study, showed serum HBsAg ≥250 IU/mL at screening, HBV DNA <20 IU/mL for ≥6 months before screening and confirmed at screening, preserved liver parameters with alanine transaminase (ALT) and aspartate aminotransferase (AST) ≤ 2 × upper limit of normal (ULN) and alkaline phosphatase (ALP), gamma-glutamyl transferase (GGT), and total bilirubin ≤ULN at screening, and preserved renal function with estimated glomerular filtration rate (eGFR) ≥70 mL/min/1.73 m^2^ at screening. Patients with a past or current diagnosis of cirrhosis, decompensation of hepatic function, liver disease other than HBV infection, or other known hepatic or biliary abnormalities, and those with severe fibrosis or cirrhosis (screening FibroScan ≥8.5 kPa) were excluded.

### Safety assessments

Safety assessments included monitoring of adverse events (AEs), laboratory tests (hematology, serum biochemistry, coagulation, urinalysis), electrocardiogram (ECG), vital signs (blood pressure, heart rate, temperature), and physical examination. Laboratory abnormalities were reported as AEs if they were deemed clinically significant by the investigator. AEs were reported until 105 days after the last dose of study treatment, graded according to severity using National Cancer Institute Common Terminology Criteria for Adverse Events version 5.0 criteria, and assessed for causality. All AEs were followed until resolution, assessed as stable, or until the subject was lost to follow-up. Non-serious AEs of special interest (NSAESI) included confirmed ALT or AST > 5 × ULN in healthy volunteers or >8 × baseline in patients, and confirmed ALT or AST > 3 × ULN in healthy volunteers or >3 × baseline in patients plus increased total bilirubin >2 × ULN or clinical jaundice. Increases in ALT or AST < 5 fold the baseline value with preserved hepatic function were not reported as serious AEs. The safety analysis was conducted in study participants who received ≥1 dose of study medication. Volunteers receiving placebo in Part 1 were pooled as one control group, as were patients receiving placebo in Part 2.

### Pharmacokinetic assessments

Plasma samples for PK analysis were collected pre-dose, at regular time points up to 24 h post-dose, and on follow-up study visits up to Day 22 in Part 1 and Day 84 after the last dose in Part 2. Urine samples were collected up to 24 h post-dose in Part 1 and up to 8 h post-dose in Part 2. A validated hybridization ELISA (hELISA) was used to determine the RO7239958 in plasma and urine samples. The capture and detection probes used in the hELISA do not discriminate between RO7239958 and the “naked” (GalNAc-deconjugated) form of the compound. Plasma PK parameters included maximum plasma concentration (*C*_max_), time to reach maximum plasma concentration (*t*_max_), area under the plasma concentration-time curve to 24 h post-dose (AUC_0–24_), or to the last measurable point (AUC_last_). Elimination of RO7239958 in urine was calculated as a percentage of the total amount administered within a 24 h period. PK parameters were calculated using non-compartmental analysis where appropriate. Selected log-transformed PK parameters were fitted as the response variables and the log-transformed dose, treated as continuous data, was fitted as a fixed effect. Dose-proportional increases in exposure to RO7239958 could be concluded if 90% confidence intervals (*CIs*) of the estimated slope of the linear regression of the log-transformed PK variable vs the log-transformed dose were within acceptance ranges.

### Population PK analysis

A population PK analysis was used to predict the concentration of RO7239958 in the liver and guide dose selection in patients. The model was built using the PK data collected in healthy volunteers. The PK profiles were best described by a three-compartment disposition open model. Based on the ASGPR-mediated delivery of RO7239958 to the liver, a saturable Michaelis-Menten liver transfer was used to describe the inter-compartmental clearance from the central to the liver. An additional compartment was used to describe the PK of RO7239958 in the liver. The covariates investigated for association with PK parameters were age, weight, sex, and race. The ability of the model to describe the data was assessed by visual inspection of goodness-of-fit plots. Relative standard errors of the parameters were also compared to measure parameter precision, as well as graphically as previously described (46). Assuming that liver exposure was the main driver of the efficacy and safety of RO7239958, the model was then used to simulate liver exposure and identify a safe dose to be tested in patients.

### Pharmacodynamic assessments

In Part 2, blood samples for the assessments of PD parameters were collected on study drug administration days (pre-dose) and on follow-up study visits up to 84 days after the last dose. Serum HBsAg was quantified using the Elecsys HBsAg II quant assay on the Cobas e601 platform (lower limit of detection 0.05 IU/mL) (Roche Diagnostics, Mannheim, Germany). Serum HBeAg was tested qualitatively using the Roche Elecsys HBeAg assay; when positive, a quantitative estimate was obtained by calibration against the Paul-Ehrlich-Institut (PEI) HBeAg reference preparation. Serum hepatitis B e antibody (anti-HBe) was tested qualitatively using the Roche Elecsys Anti-HBe assay. Plasma HBV DNA was measured using the Roche Cobas HBV Test assay (lower limit of quantification 10 IU/mL).

### Immunogenicity

Immunogenicity samples were taken to assess the presence of anti-drug antibodies (ADAs) in Part 1 (pre-dose Day 1 and follow-up Days 22 and 85) and Part 2 (pre-dose Days 1 and 29 and follow-up Day 57).

### Statistical analysis

Descriptive statistics were used to summarize categorical variables as proportions and continuous variables as mean ± standard deviation (SD). The PD analysis included patients who received ≥1 dose of study medication with ≥1 post-treatment assessment; changes in serum PD parameters over time were plotted for individual patients. Population PK analysis and simulations were performed with NONMEM version 7.4.1 (ICON Development Solutions, USA). All analysis data sets were created using SAS version 9.4. Graphical analyses were performed using R version 3.5.1 and prediction-corrected visual predictive check (pcVPC) analysis used Perl speak to NONMEM (PsN 4.4.8).

### Highlights

- RO7239958 is a liver-targeted locked nucleic acid (LNA) antisense oligonucleotide (ASO) that induces the intrahepatic degradation of PAPD5 and PAPD7 mRNA.
- In patients with chronic hepatitis B, two subcutaneous doses of RO7239958 (0.2 and 0.4 mg/kg) administered at a 4-week interval were safe and well-tolerated, achieving modest pharmacodynamic effects.
- Although RO723995 demonstrated a narrow therapeutic window, PAPD5 and PAPD7 remain potential targets of interest for inhibiting HBV expression.

### Impact and implications

Hepatitis B virus (HBV) establishes a persistent infection as a covalently closed circular DNA (cccDNA) in the nucleus of infected liver cells. Inhibition of host factor poly(A) RNA polymerases D5 and D7 (PAPD5 and PAPD7) with small-molecule agents has been shown to reduce hepatitis B surface antigen (HBsAg) levels in infected liver cells without affecting cell viability. RO7239958 is an HBV gene expression inhibitor designed to target the liver and induce degradation of PAPD5 and PAPD7 mRNA. This first-in-human clinical study of RO7239958 demonstrated a narrow therapeutic window due to a risk of hepatotoxicity at higher doses. However, targeting PAPD5 and PAPD7 remains a viable therapeutic strategy in the search for an HBV cure.

## Data Availability

Data availability statement: For eligible studies, qualified researchers may request access to individual patient level clinical data through a data request platform. At the time of writing this request platform is Vivli. https://vivli.org/ourmember/roche/. For up to date details on Roche's Global Policy on the Sharing of Clinical Information and how to request access to related clinical study documents, see here: https://go.roche.com/data_sharing. Anonymized records for individual patients across more than one data source external to Roche can not, and should not, be linked due to a potential increase in risk of patient re-identification.

## SUPPLEMENTAL MATERIAL

Supplemental material aac.00679-25-s0001.docx Table S1; Fig. S1.

ASM does not own the copyrights to Supplemental Material that may be linked to, or accessed through, an article. The authors have granted ASM a non-exclusive, world-wide license to publish the Supplemental Material files. Please contact the corresponding author directly for reuse.
