# Supplementary material for: Safety, pharmacokinetics, and pharmacodynamics of the antisense oligonucleotide RO7239958 in healthy volunteers and adults with chronic hepatitis B infection
Source: Antimicrob Agents Chemother. 2025 Nov 4;69(12):e00679-25. doi: 10.1128/aac.00679-25 (PMC12691668; doi:10.1128/aac.00679-25)

**Supplementary Table 1.** Results of investigations performed in a healthy volunteer with a grade 3

alanine transaminase elevation considered related to study drug

| Study day | Test^a^ | | | | | |
| --- | --- | --- | --- | --- | --- | --- |
|  | ALT U/L  (0-45) | AST U/L  (0-45) | ALP U/L  (40-110) | GGT U/L (0-60) | Tot. Bilirubin μmol/L (0-25) | Other^b^ |
| -1 | **64** | 32 | 95 | 9 | 10 |  |
| 2 | **63** | 29 | 89 | 13 | 8 |  |
| 8 | **93** | 43 | **124** | 13 | 7 |  |
| 15 | **154** | **57** | **117** | 15 | 10 |  |
| 22 | **298** | **78** | **139** | 21 | 9 |  |
| 24 | **336** | **80** | **168** | NA | NA |  |
| 31 | **355** | **79** | **120** | 32 | 12 | HAV, HEV, CMV, EBV serology negative |
| 40 | **244** | **57** | **124** | NA | 8 |  |
| 43 | **240** | **55** | **115** | 41 | 11 |  |
| 50 | **199** | 43 | **123** | 44 | 11 | Serum ceruloplasmin normal Serum copper 8 μmol/L^c^ |
| 58 | **161** | 45 | 85 | 39 | 9 |  |
| 63 | NA | 42 | **122** | 33 | 7 |  |
| 65 | NA | NA | NA | NA | NA | Liver biopsy: Mild steatohepatitis;  liver copper level 24 μg/g^d^ |
| 71 | **127** | 38 | **125** | 29 | 7 |  |
| 78 | **138** | 42 | 108 | 25 | 7 |  |
| 85 | **117** | 38 | 94 | 23 | 11 |  |

^a^Reference ranges indicated in brackets; ^b^Participant was reported to have tested serologically negative for HCV, HIV and Syphilis; ^c^Reference range 11.8-22.8 μmol/L; ^d^Normal <50 μg/g.

Abbreviations: ALT, alanine transaminase; ALP, alkaline phosphatase; AST, aspartate aminotransaminase; CMV, cytomegalovirus; EBV, Epstein-Barr virus; GGT, gamma-glutamyl transferase; HAV, hepatitis A virus; HEV, hepatitis E virus; HCV, hepatitis C virus; HIV, human immunodeficiency virus; NA, not available.

**Supplementary Figure 1.** Predicted liver concentration of RO7239958 versus time at dosing levels of 0.2 and 0.4 mg/kg every 4 weeks. The dashed red line represents the predicted median liver exposure at no-observed-adverse-effect level (NOAEL) in non-human primates (NHPs) (data on file). The dashed blue lines represent the predicted median liver exposure at Cmax after single dose of 1 and 1.5 mg/kg in healthy volunteers. The shaded area represents the 90% prediction interval.


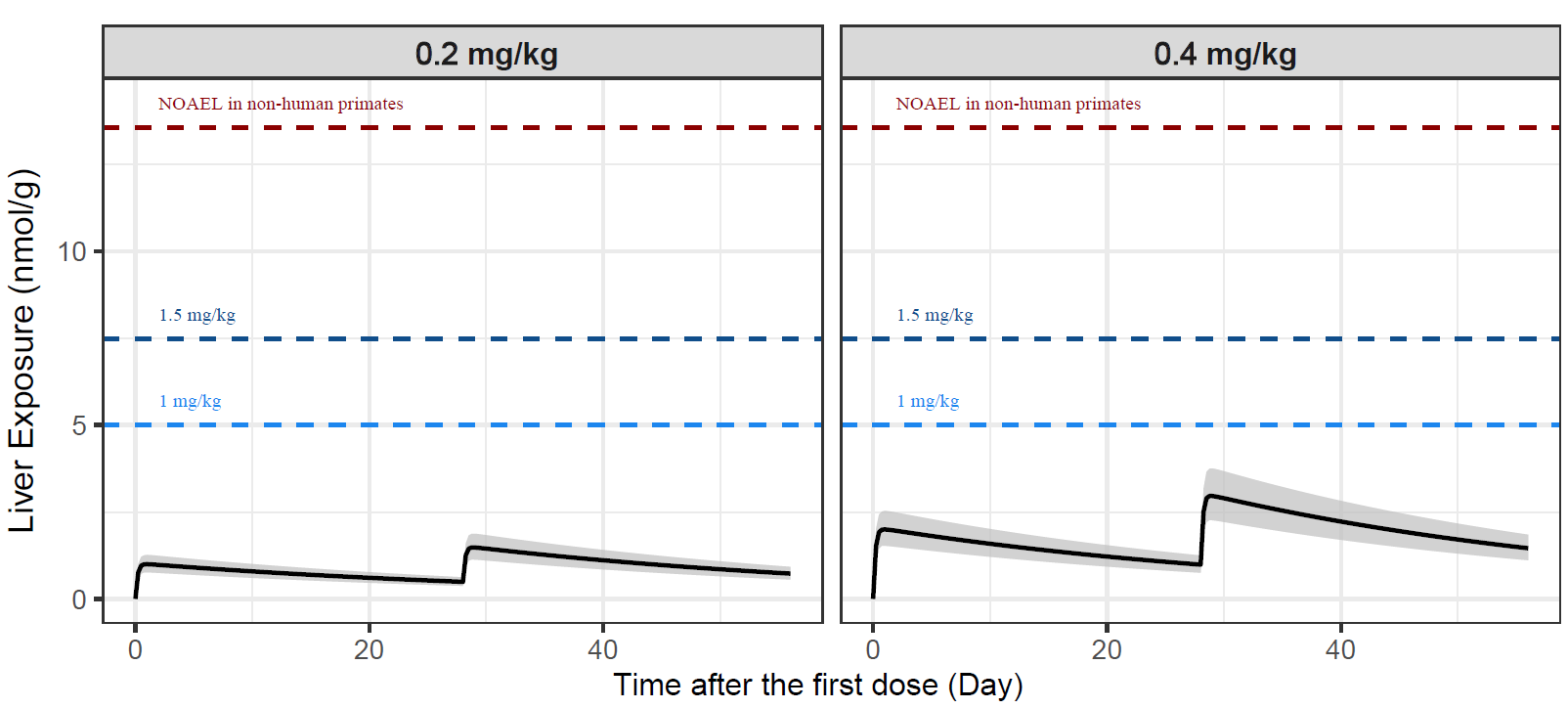

Supplement: Supplemental material — Table S1; Fig. S1. [file aac.00679-25-s0001.docx]
